# Study on the Effect of Electron/Hole Injection on the Energy-Storage Properties of Polymer Dielectrics

**DOI:** 10.3390/polym16192750

**Published:** 2024-09-28

**Authors:** Guang Liu, Yuhao Chen, Yang Cui, Lifang Shen, Taiquan Wu, Chen Chen, Yunxia Luo, Shubin Yan

**Affiliations:** 1 Nanxun Innovation Institute, Zhejiang University of Water Resources and Electric Power, Hangzhou 310018, China; fusuheihei@163.com (Y.C.); cuiy@zjweu.edu.cn (Y.C.); shenlf@zjweu.edu.cn (L.S.); wutq@zjweu.edu.cn (T.W.); luoyx@zjweu.edu.cn (Y.L.); yanshb@zjweu.edu.cn (S.Y.); 2 School of Electrical Engineering, Zhejiang University of Water Resources and Electric Power, Hangzhou 310018, China

**Keywords:** polymer dielectric, asymmetric, electron injection, barrier, energy-storage characteristics

## Abstract

As a critical component of electrostatic capacitors, the polymer dielectric directly affects the performance of the capacitor. In this work, Polycarbonate (PC)/Polyvinylidene fluoride (PVDF) asymmetric bilayer polymer dielectrics were prepared, and the influence of different polymer materials’ barrier characteristics on various electrical properties of composite dielectrics was studied by changing the direction of applied electric fields. Research has found that the dielectric constant of a composite dielectric is between PVDF and PC (approximately 4.8 at 10 Hz) and is independent of the relative position of PVDF and PC in the dielectric. However, the relative position of PC and PVDF has a significant impact on the energy-storage characteristics of composite dielectrics. When PVDF comes into contact with the negative electrode, even though PC has a higher hole barrier, the composite dielectric can only withstand a maximum electric-field strength of 400 MV/m, which is much lower than the maximum electric-field strength that pure PC can withstand (520 MV/m), and it only achieves an energy-storage density of 3.7 J/cm^3^. When the PC comes into contact with the negative electrode, the high electron barrier of the PC effectively suppresses the injection of electrons at the electrode. It can withstand the same electric-field strength as PC (520 MV/m), achieving an energy-storage density of 5.48 J/cm^3^, which is 1.46 times that of pure PC and 1.64 times that of PVDF. This experiment effectively combined the advantages of PC and PVDF by utilizing the electron/hole barrier of polymer materials to obtain a fully organic dielectric with excellent energy-storage performance.

## 1. Introduction

As important energy-storage devices with ultra-high power densities, electrostatic capacitors are widely used in electronic and electrical systems such as electric vehicles, grid-connected photovoltaics, medical defibrillators, and oil-exploration drilling rigs [1,2,3]. Polymer dielectrics have many advantages, such as good processability, high reliability, and high breakdown strength, and are an important component of electrostatic capacitors. Therefore, the development of polymers with excellent electrical properties is of great significance in improving the performance of electrostatic capacitors [4,5]. The energy-storage density of a dielectric is directly related to its dielectric constant and the applied electric-field intensity. A large dielectric constant is conducive to obtaining the high polarization intensity of the dielectric, thereby obtaining the ideal charge energy density [6,7]. PVDF has attracted much attention due to its higher dielectric constant compared to other polymer materials. However, its poor insulation leads to a large number of carriers injected under high electric fields, resulting in increased conductivity loss and reduced charge–discharge efficiency, seriously affecting its energy-storage characteristics [8,9,10].

**Figure 1:**
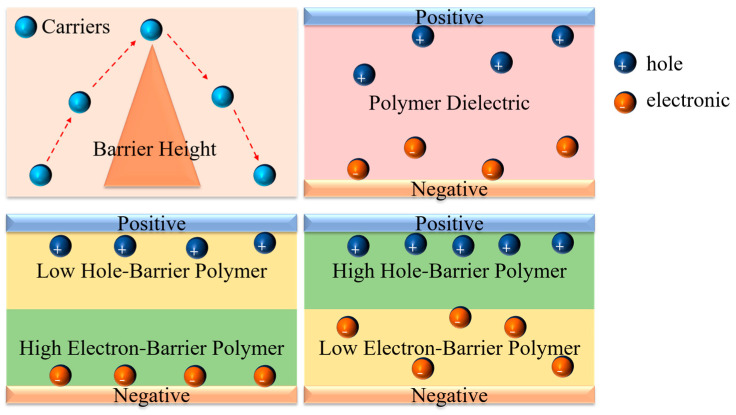
Theoretical schematic diagram.

The energy-storage density of a dielectric film is proportional to the square of the applied electric-field intensity. Therefore, the breakdown of the dielectric is the most important factor affecting its ability to achieve a high energy-storage density. The breakdown of the dielectric is mainly caused by the injection of carriers (electrons and holes) at the electrode under high electric fields, causing the growth of electrical tree branches, thereby creating conductive pathways and causing the breakdown of the dielectric [11,12,13,14]. There are few studies on the impact of the injection of electrons and holes on dielectric energy storage. Carriers at the electrode need to cross the electron/hole barrier of the dielectric itself before they can be injected into the dielectric. Different dielectrics have different electron/hole barrier heights, which have different effects on the injection of electrons/holes at the electrode. By adjusting the position of different polymers, a synergistic suppression of electrons and holes at the electrode can be achieved. The theoretical schematic diagram is shown in Figure 1.

As a typical ferroelectric polymer, PVDF has a high dielectric constant (~10), while linear polymers, such as polycarbonate, polyetherimide, etc., have excellent insulating properties. The aforementioned polymers have different electron/hole barrier heights [15,16,17]. Using different polymers to prepare asymmetric-structure composite films and changing the direction of the electric-field application is an effective means to study the impact of electron and hole injection on the breakdown and energy-storage properties of the composite dielectric.

Based on the theory mentioned above, this research work led to the preparation of an all-organic composite dielectric with an asymmetric structure by combining PVDF with PC film. By adjusting the direction of the applied electric field during the test, the influence of electron and hole injection on the breakdown and energy-storage characteristics of the composite dielectric was explored. Research has found that when the PVDF layer is in contact with the negative electrode, the composite dielectric can withstand an electric-field intensity of 400 MV/m and obtain an energy-storage density of 3.71 J/cm^3^ with a charge–discharge efficiency of 92.65%. When the PC layer is in contact with the negative electrode, the composite dielectric can withstand an electric–field intensity of up to 520 MV/m and obtain an energy-storage density of 5.49 J/cm^3^, and the charge–discharge efficiency is still as high as 89.88%. The experimental results have a certain guiding significance for the structural-design optimization, insulation performance and energy-storage performance improvement of polymer dielectrics.

## 2. Experimental

### 2.1. Raw Materials

The chemical raw materials and chemical reagents used in the experiment mainly include PVDF powder (Shanghai Huayi 3 F New Materials Co., Ltd., Shanghai, China, Purity > 99%), PC particles (PolyK Technologies, State College, PA, USA), N-N dimethylformamide (Sinopharm Chemical Reagent Co., Ltd., Shanghai, China, Analytical purity), and tetrahydrofuran (Shanghai Aladdin Biochemical Technology Co., Ltd., Shanghai, China, Standard for GC, purity > 99.9%). These raw materials and chemical reagents were used directly after purchase without further processing.

### 2.2. Preparation of Composite Films

Firstly, 1 g of PVDF powder was added to a 10 mL N-N dimethylformamide solution and stirred continuously at room temperature for 10 h using a magnetic stirrer to dissolve the PVDF completely. A vacuum oven was used to remove tiny bubbles from the PVDF solution. Subsequently, the PVDF solution was evenly coated on a clean glass plate, and the PVDF colloid was kept in a regular oven and a vacuum oven at 80 °C for 10 h to allow the solvent to evaporate fully, ultimately obtaining the required PVDF film.

Similarly, 1.5 g of PC particles were dissolved in a tetrahydrofuran solution, and the aforementioned operation process was used to obtain the required PC film. Finally, the PVDF/PC asymmetric-structure fully organic composite dielectric was obtained by hot pressing at 130 °C and 10 MPa pressure for 5 min.

### 2.3. Structural Characterization and Electrical Performance Testing

In the experiment, an X-ray diffractometer (EMPYREAN, Almelo, The Netherlands) and a scanning electron microscope (HITACHIS-3400 N, Tokyo, Japan) were used to characterize and analyze the phase composition, thickness and internal defects of the dielectric thin film. Coating the polymer surface with metal electrodes is necessary to conduct relevant electrical performance tests. In this experiment, we first sandwiched the polymer film in a symmetrical mask plate containing holes with a diameter of 3 mm. Subsequently, a polymer dielectric with a surface containing gold electrodes was obtained by continuously operating it for 100 s at a power of 45 W using a plasma magnetron sputter coater (SC-SU-I, SETCAS Electronics Co., Ltd., Beijing, China).

The dependence of the dielectric constant of dielectric thin films on frequency was tested and analyzed using a broadband dielectric spectrometer (DMS1000, Partulab BALAB Technology Co., Ltd., Wuhan, China). The current density and hysteresis loop of dielectric thin films under different electric-field intensities were tested using a ferroelectric comprehensive testing system (Polyk CPE1901), and the energy-storage density and charge–discharge efficiency of dielectric thin films under different electric-field intensities were calculated.

## 3. Results and Discussion

**Figure 2:**
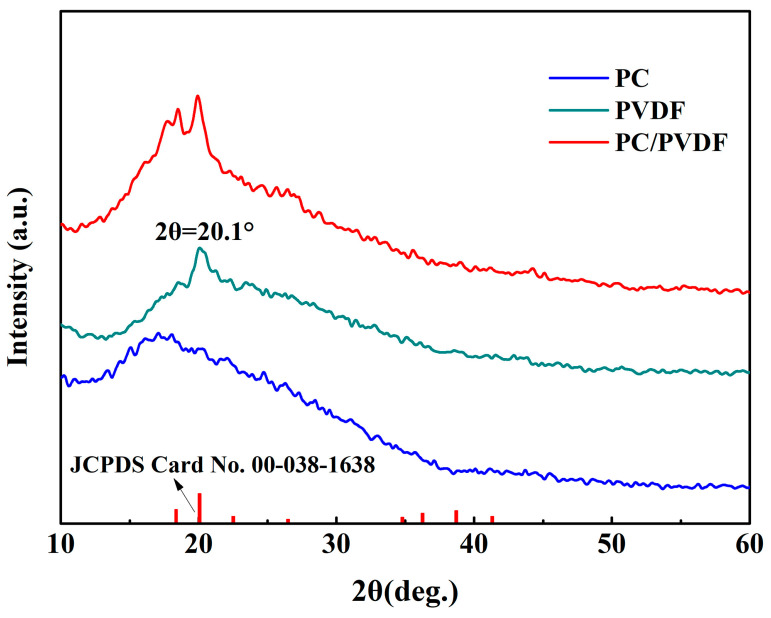
XRD patterns of PC, PVDF, and PC/PVDF.

Asymmetric PC/PVDF composite films were prepared with solution casting combined with hot pressing. In order to prove the composition of the prepared composite films, X-ray diffractometer (XRD) tests were conducted on the dielectric thin films, and the test results are shown in Figure 2. It can be seen that the characteristic peak of PVDF is 2θ = 20.1°, which corresponds to the standard card (JCPDS Card No. 00-038-1638). PC shows a broad peak near 17°, which is consistent with literature reports [18,19]. In addition, no other characteristic peaks are present, indicating that the prepared PC and PVDF are pure. The characteristic peaks of PC and PVDF simultaneously appeared in the PC/PVDF composite film, and there are no other new characteristic peaks. This proves that the composite film is only a physical composite of PC and PVDF without the generation of new phases.

**Figure 3:**
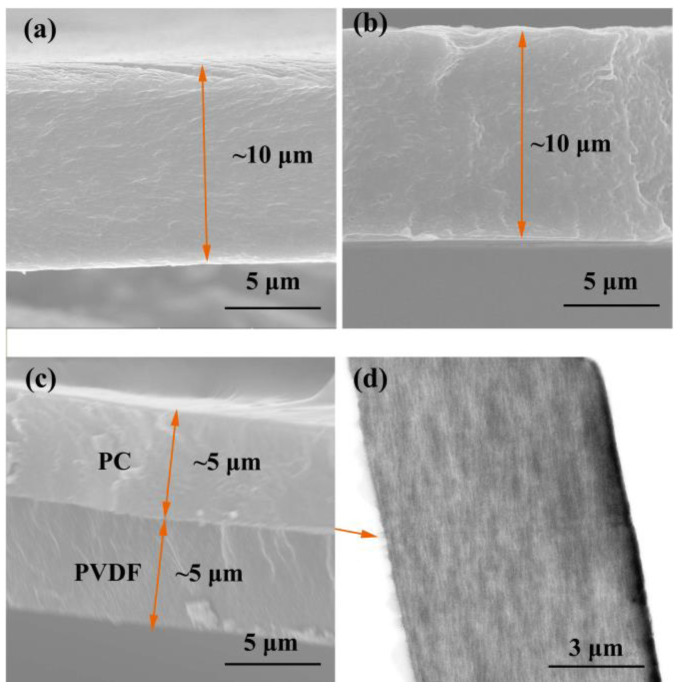
Characterization of the cross-sectional structure of dielectric thin films. SEM images of the cross-section of dielectric thin films, (**a**) PVDF, (**b**) PC, (**c**) PC/PVDF; TEM image of the cross-section of PC/PVDF (**d**).

The cross-section of the dielectric film was observed through a scanning electron microscope (SEM) and transmission electron microscope (TEM) to analyze the thickness and internal defects of the dielectric film. The test results are shown in Figure 3. From the figure, it can be seen that the thickness of the single-layer dielectric film is about 10 μm. The cross-section of the polymer film is flat and smooth, without obvious defects such as pores, indicating that the prepared dielectric film has good quality. Figure 3c is a cross-sectional SEM image of a PC/PVDF composite dielectric. It can be seen that there is a clear boundary line in the composite film, which proves that the composite film is a double-layer composite dielectric. The thickness of both the PVDF layer and the PC layer in the composite dielectric is about 5 μm, and the total thickness is consistent with the thickness of the single-layer PVDF and PC in Figure 3a,b. Moreover, no obvious defects, such as gaps and holes, are observed at the interface in the SEM test results, indicating that the double-layer film has good bonding and interface quality.

In order to further observe the interface quality of the composite dielectric, TEM testing was conducted on the cross-section of the composite dielectric, and the results are shown in Figure 3d. From the TEM test results, it can be seen that the composite dielectric is composed of two layers with a clear boundary (as indicated by the arrow in the figure). In the test results, it can be seen that the two polymers are tightly bonded, and there are almost no defects, such as gaps or voids at the interface. This strongly proves that the interface quality of the composite dielectric is good, which lays the foundation for obtaining good electrical properties of the composite dielectric.

**Figure 4:**
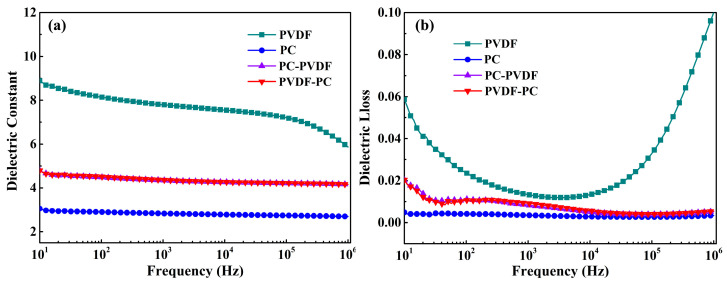
Frequency dependence of dielectric properties of dielectric thin films. (**a**) Dielectric constant; (**b**) Dielectric loss.

The dielectric constant of materials directly affects their polarization intensity and has an important impact on their energy-storage characteristics [20,21]. The frequency dependence of the dielectric constant and dielectric loss of the polymer film was tested through broadband dielectric spectroscopy, and the results are shown in Figure 4. PC is a typical linear polymer: its dielectric constant is almost unaffected by frequency changes and remains around three over a wide frequency range, and the same is true for dielectric loss. PVDF is a typical ferroelectric polymer, and its dielectric constant shows a gradually decreasing trend as the frequency increases.

In addition, the impact of different test directions on the dielectric properties of PC/PVDF double-layer composite films is mainly explored. In Figure 4, PVDF-PC represents that the PC layer in the composite dielectric is in contact with the negative electrode during testing, and PC-PVDF is the opposite. It can be seen from the figure that the dielectric constant of the composite film is between PVDF and PC and is much lower than that of PVDF. Besides, it can be found that the dielectric constant of the composite film at the same frequency is not affected by the testing direction. This is because of the alternating current applied by the broadband dielectric spectroscopy test: the direction of the electric field is alternating, so the testing direction of the composite film has no effect on the dielectric properties. Figure 4b shows the variation of dielectric loss with frequency for several dielectrics. It can be seen that the dielectric loss of PC is the lowest (only about 0.004). The dielectric loss of PVDF is relatively significant. At 10 Hz, its loss value is 0.058, which is 14.5 times that of PC, and it changes greatly with frequency. The dielectric loss of composite dielectric is between PC and PVDF.

In order to better understand the polarization in composite films, the dielectric constant of composite films was calculated according to the following formula [9,22]:

(1) $$\frac{1}{\varepsilon }=\frac{x_{1}}{\varepsilon_{1}}+\frac{x_{2}}{\varepsilon_{2}}$$

**Figure 5:**
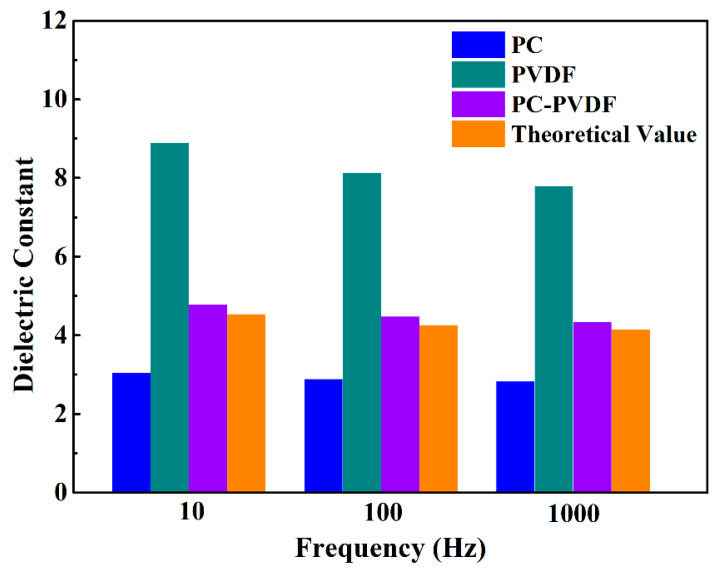
Test values of the dielectric constant of dielectric thin films and theoretical calculation values of composite thin films.

In the formula, *ε* is the dielectric constant of the PC/PVDF composite dielectric, and *ε*_1_ and *ε*_2_ represent the dielectric constants of PC and PVDF, respectively. *x*_1_ and *x*_2_ are the volume fractions of the PC layer and PVDF layer in the composite film, respectively. The test values of the dielectric constant of PC, PVDF, and double-layer composite materials at 10 Hz, 100 Hz, and 1000 Hz, as well as the theoretical calculation values of the dielectric constant of composite thin films, are shown in Figure 5.

It can be seen that the test value of the dielectric constant of the composite film is slightly higher than the theoretical calculation value. That is because the accumulation of interface charges at the heterogeneous interface in the layered film can enhance interface polarization, thereby increasing the dielectric constant of the composite dielectric [23,24]. However, the theoretical calculation did not consider the effect of interface polarization on the dielectric constant of the composite film. In composite films, the presence of PC can capture the motion space of PVDF molecular chains, thereby reducing the dielectric constant of the composite film [24]. Therefore, the measured dielectric constant of the composite film is only slightly higher than the theoretical calculation value.

**Figure 6:**
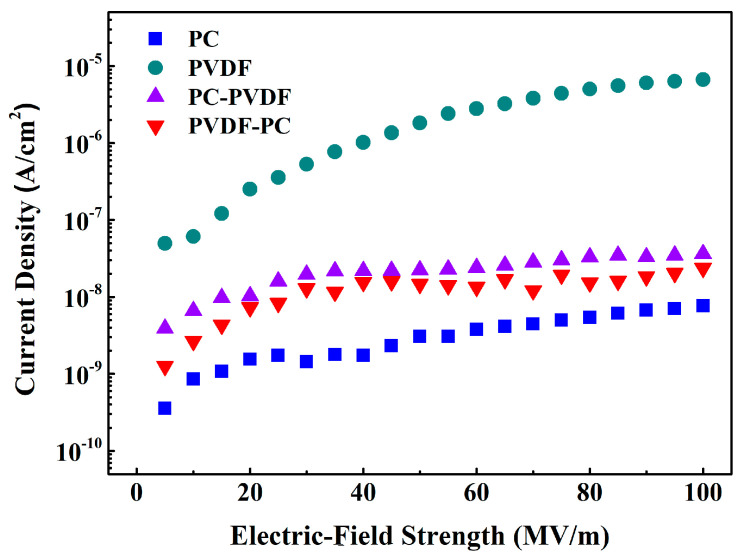
Current density of dielectric thin films under different electric-field intensities.

In order to investigate the effect of different test electric-field directions on the internal current density of composite thin films, the current density of composite dielectrics under different electric fields was tested, and the test results are shown in Figure 6. It can be seen that the current density of PVDF is the highest, which also reflects its poor insulation performance. In our previous research work, we found that the electron-barrier height and hole-barrier height of PVDF are 0.79 eV and 1.42 eV, respectively, making it easier for carriers to inject into the film, resulting in a higher current density [9]. On the contrary, PC has a high electron-barrier height of 1.44 eV and a hole-barrier height of 2.27 eV, respectively, making it difficult for carriers to inject into the interior of the PC film [9]. Therefore, the current density of the PC is relatively low, which also indicates its good insulation performance.

The current density of the composite film composed of PC and PVDF at various electric-field strengths is between that of PC and PVDF, and for the composite film, changing the direction of the applied electric field does make a difference in the current density of the composite film. Compared with PVDF, PC not only has a higher electron barrier but also has a higher hole barrier [9]. When PC comes into contact with the negative electrode, its high electron barrier can effectively suppress electron injection at the negative electrode, while when PC comes into contact with the positive electrode, its high hole-barrier height can suppress hole injection at the positive electrode. By comparing the current densities of the two, it can be found that the current density of the composite film is lower when PC comes into contact with the negative electrode, which indicates that the electronic barrier height of dielectric thin films has a more important impact on its current density.

**Figure 7:**
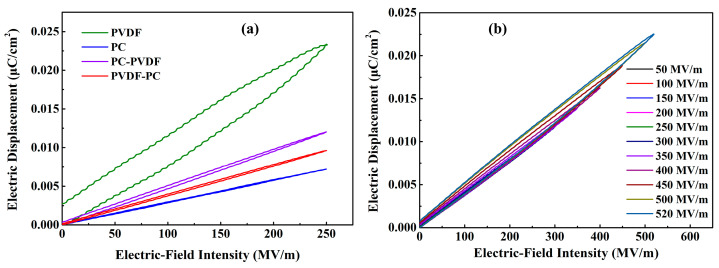
Hysteresis loop diagram of dielectrics. (**a**) Electric hysteresis loops of several dielectrics under an electric field of 250 MV/m; (**b**) Electric hysteresis loops of PVDF-PC under different electric-field strengths.

Finally, the hysteresis loops of polymer dielectric under different electric-field intensities were tested using a ferroelectric comprehensive tester to analyze the energy-storage characteristics of the dielectric thin film, and the results are shown in Figure 7. The hysteresis loop reflects the relationship between the polarization intensity (*D*) of the dielectric material and the electric field (*E*), and this relationship can be expressed as $D=\varepsilon_{1}\varepsilon_{2}E$ [23]. Therefore, under the same electric-field intensity, the larger the dielectric constant of the dielectric material, the better it is to obtain a high polarization intensity, thus achieving an excellent charging energy density. Based on the aforementioned data, the charging energy density (*U*_c_) and discharging energy density (*U*_d_, referred to as energy-storage density *U* in this article) of the dielectric under a specific electric-field strength can be calculated using U=∫EdD [24]. On this basis, the charge–discharge efficiency (*η*) of the dielectric under a certain electric-field strength can be calculated using $\eta =\frac{U_{d}}{U_{c}}$ [24].

Figure 7a shows the hysteresis loops of several dielectric films under an electric-field strength of 250 MV/m. As can be seen from the figure, PVDF has the maximum electric displacement polarization, and PC has the minimum, while the polarization intensity of composite dielectrics is between the two. In particular, the electric displacement polarization of PVDF-PC is significantly lower than that of PC-PVDF. This is because when a unidirectional electric field is applied, the PC can effectively suppress electron injection, which can effectively reduce interface charge accumulation and interface polarization. Therefore, it has a lower polarization intensity. In addition, it can be seen that the gap between the charge and discharge curves of PVDF is the largest, which means it has greater energy loss and the lowest charge–discharge efficiency, which is directly related to its large current density. In addition, the gap between the charge and discharge curves of PVDF-PC is very similar to that of PC, significantly smaller than PC-PVDF. This indicates that when PC comes into contact with the negative electrode, it effectively suppresses the injection of electrons at the electrode, reduces the current density of the dielectric, and improves its charge–discharge efficiency. This also reflects that electron injection has a more significant impact on the energy-storage characteristics of dielectrics. PVDF-PC has thinner charge and discharge curves under various electric-field strengths, which means it has lower energy loss and higher charge–discharge efficiency, as shown in Figure 7b.

**Figure 8:**
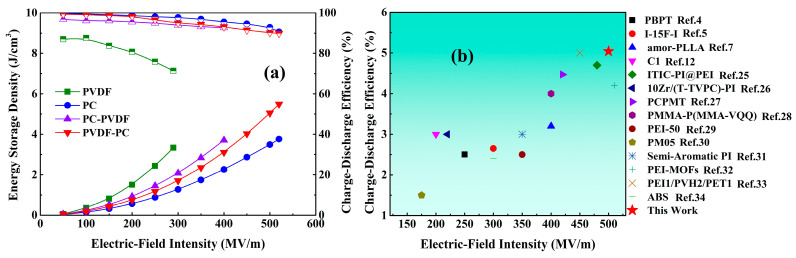
Energy-storage properties of dielectric films. (**a**) Energy-storage density and charge–discharge efficiency of dielectric films under different electric fields; (**b**) Comparison of the energy-storage characteristics of this research work with related research work when the charge and discharge efficiency exceeds 90% [4,5,7,12,25,26,27,28,29,30,31,32,33,34].

The energy-storage density and charge–discharge efficiency of the dielectric thin film under different electric-field intensities were calculated by integrating the hysteresis loop, as shown in Figure 8a. From the figure, it can be seen that under the same electric-field intensity, PVDF has the highest energy-storage density and the lowest charge–discharge efficiency, while PC has the lowest energy-storage density but the highest charge–discharge efficiency, which is consistent with the analysis and speculation mentioned above. In comparison, under the same electric-field intensity, the PC/PVDF composite film has an ideal energy-storage density and charge–discharge efficiency. When PVDF is in contact with the negative electrode (PC-PVDF), carriers are more easily injected into the dielectric, which can cause carriers to accumulate at the interface, thereby enhancing interface polarization. Therefore, its energy-storage density is higher than that of PVDF-PC.

**Figure 9:**
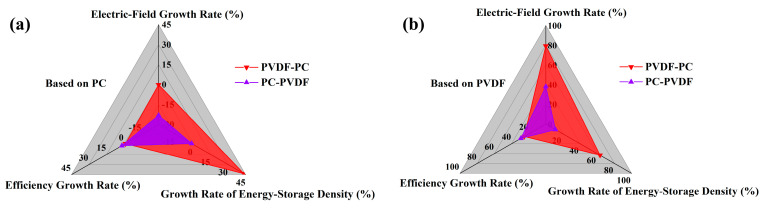
Growth rate of energy-storage properties of the PC/PVDF composite dielectric. (**a**) Relative to PC; (**b**) Relative to PVDF.

In addition, when PVDF is in contact with the negative electrode (PC-PVDF), carriers are easily injected into the dielectric, resulting in a higher current density and poor insulation performance. It can only withstand a maximum electric-field strength of 400 MV/m, which is lower than the maximum electric-field strength that PVDF-PC can withstand (520 MV/m). Although PC has a high hole-barrier height and can effectively suppress hole injection, the faster electron-migration rate causes the breakdown of the dielectric, which is mainly caused by electron migration. Therefore, when PVDF comes into contact with the negative electrode, the current density of the composite dielectric increases and the insulation strength slightly deteriorates. When the PC is in contact with the negative electrode, the high electron-barrier height of the PC can effectively suppress electron injection. Even in the presence of PVDF, the composite film can still withstand the same electric-field intensity as PC (520 MV/m), and under this electric-field intensity, the composite film achieved an energy-storage density of 5.49 J/cm^3^, which is 46.01% higher than that of pure PC, and its charge–discharge efficiency can still be maintained at 89.86%, which is only 0.85% lower than that of pure PC. Under the condition that the charge–discharge efficiency is higher than 90%, the composite film can withstand an electric-field intensity of 500 MV/m and obtain an energy-storage density of 5.04 J/cm^3^. This energy-storage characteristic has significant advantages compared to recent research work, as shown in Figure 8b. In addition, the growth rate of various energy-storage properties of the composite film compared with pure PC and pure PVDF was calculated, as shown in Figure 9.

Compared with pure PC, the maximum electric-field intensity that PC-PVDF and PVDF-PC can withstand is increased by −23.07% and 0%, respectively, and their energy-storage density and charge–discharge efficiency are increased by −1%, 43.6%, 2.2% and −0.85% respectively, as shown in Figure 9a. Overall, PC has a high electron barrier, which can effectively suppress electron injection when in contact with the negative electrode. Based on this, combined with the high dielectric constant of PVDF, the breakdown and energy-storage characteristics of polymer dielectrics have been synergistically optimized. Compared with pure PVDF, the growth rate of PVDF-PC in terms of energy-storage density and the maximum electric field it can withstand is significantly better than that of PC-PVDF, as shown in Figure 9b.

## 4. Conclusions

In this work, an asymmetric-structure PC/PVDF double-layer all-organic composite film was prepared. By adjusting the direction of electric-field application, the influence of electron and hole injection on various electrical properties of dielectric was studied in depth. By testing the relative dielectric constant, leakage current, and energy-storage characteristics of the dielectric, it was found that different electric-field directions have an important impact on the electrical properties of the dielectric. When PC is in contact with the positive electrode, although its large hole-barrier height can effectively suppress hole injection at the electrode, the low electron-barrier height of PVDF makes it easier for electrons to be injected into the dielectric, and ultimately the various electrical properties of the composite film performance are not ideal. When PC is in contact with the negative electrode, its large electron-barrier height can more effectively suppress electron injection at the electrode. Combined with the high dielectric constant of PVDF, the composite dielectric can withstand an electric-field intensity of 520 MV/m, achieving an energy-storage density of 5.49 J/cm^3^, which is 1.46 times that of pure PC and 1.65 times that of pure PVDF, and has a charge–discharge efficiency of nearly 90%.

## Data Availability

Data is contained within the article.
